# Late Recognition of Glucose-6-Phosphate Dehydrogenase Deficiency in a 70-Year-Old Man: A Case Report

**DOI:** 10.7759/cureus.108227

**Published:** 2026-05-04

**Authors:** Julie Huynh, Lauren Eisenbud, Rodolfo Gutierrez, Fukai L Chuang, Daniel Dichter

**Affiliations:** 1 Hematology and Medical Oncology, University of California Los Angeles, Los Angeles, USA; 2 Palliative Care Medicine, University of California Los Angeles, Los Angeles, USA

**Keywords:** acute hemolytic anemia, fava beans, glucose-6-phosphate-dehydrogenase deficiency (g6pd), macrocytosis, sulfa drug

## Abstract

Glucose-6-phosphate dehydrogenase (G6PD) deficiency is the most common enzymatic disorder of red blood cells worldwide and typically presents in childhood or early adulthood following exposure to oxidative stressors. Late-life diagnosis is uncommon and may be overlooked in patients without exposure to classic hemolytic triggers. We report the case of a 70-year-old man with intermittent, mild macrocytic anemia who was ultimately diagnosed with G6PD deficiency. This case highlights how G6PD deficiency may remain clinically silent for decades, discusses reasons for delayed diagnosis, and emphasizes the importance of considering inherited hemolytic disorders in older adults with unexplained macrocytic anemia.

## Introduction

G6PD deficiency is an X-linked recessive disorder affecting more than 500 million people globally, occurring most often in male individuals of African, European, Middle Eastern, and Asian descent [1]. The enzyme G6PD plays a critical role in protecting red blood cells from oxidative damage by maintaining reduced glutathione levels. Deficiency predisposes erythrocytes to hemolysis when exposed to oxidative stressors such as infections, certain medications, and fava beans. Classic presentations include neonatal jaundice or acute hemolytic anemia in younger individuals [2]. In the absence of triggers, many patients remain asymptomatic and therefore, undiagnosed well into adulthood. Recognition in elderly patients is rare and may be confounded by more common causes of anemia in this age group.

Although G6PD deficiency is the most frequently encountered red cell enzymopathy, it is not the only inherited enzymatic cause of hemolysis that may present outside childhood. Disorders such as pyruvate kinase deficiency and, more rarely, hexokinase or other glycolytic enzyme defects can also produce Coombs-negative hemolysis, but these conditions usually declare themselves earlier in life and are often accompanied by longstanding anemia or splenomegaly [3]. In contrast, G6PD deficiency may remain clinically unapparent for decades and only become evident after exposure to oxidant medications, acute illness, or other metabolic stressors encountered later in adulthood [1,4]. For this reason, episodic hemolysis in older patients without a prior hematologic diagnosis should still prompt consideration of G6PD deficiency alongside other enzymopathies, particularly when the presentation is triggered by a recognizable oxidative exposure.

## Case presentation

A 70-year-old man was referred to the Hematology Department on December 22, 2025, for evaluation of intermittent mild anemia noted for several years. His hemoglobin values ranged from 12 to 13 g/dL (maximum 14 g/dL), with varying degrees of macrocytosis (Table 1). He reported intermittent mild fatigue but denied jaundice, dark urine, bleeding, weight loss, or constitutional symptoms. His past medical history only included hypothyroidism, and no known personal or family history of hemolytic anemia. He denied prior episodes of severe anemia or fatigue requiring medical evaluation. 

**Table 1:** Laboratory values showing intermittent and varying degrees of macrocytosis and anemia

Component (Reference Range)	February 25, 2022	October 10, 2022	January 12, 2023	August 25, 2023	September 17, 2024	September 16, 2025	December 22, 2025
White Blood Cell Count (3.98 - 10.04 thousand/uL)	6.75	4.45	5.25	5.70	6.98	4.42	4.08
Hemoglobin (13.7 - 17.5 g/dL)	13.3 (low)	13.0 (low)	14.0	12.7 (low)	14.0	12.6 (low)	13.0 (low)
Mean Corpuscular Volume (79.0 - 94.8 fL)	100.9 (high)	102.5 (high)	103.2 (high)	100.3 (high)	100.7 (high)	99.0 (high)	99.8 (high)
Platelet Count (163 – 369 thousand/uL)	292	271	262	246	246	250	238

Initial laboratory evaluation demonstrated normal iron studies, vitamin B12, and folate levels. Reticulocyte count was intermittently mildly elevated. Lactate dehydrogenase was at the upper limit of normal, haptoglobin was low-normal, and indirect bilirubin was intermittently mildly elevated, suggesting low-grade hemolysis. Direct antiglobulin testing was negative.

Given the evidence of episodic macrocytosis and likely hemolysis without an autoimmune etiology, enzymatic testing was pursued. Quantitative G6PD assay revealed decreased enzyme activity at 14%, consistent with G6PD deficiency. The patient was counseled on the avoidance of triggering medications and foods, possible infection-related hemolysis, and advised to inform healthcare providers of his diagnosis. The patient then reported frequently eating fava beans and recalled that some family members were not allowed to eat them. After avoiding fava beans for three months, his hemoglobin became normocytic and increased to 14 g/dL (Table 2).

**Table 2:** Laboratory values after three months abstaining from fava beans

Component (Reference Range)	March 26, 2026
White Blood Cell Count (3.98 - 10.04 thousand/uL)	5.85
Hemoglobin (13.7 - 17.5 g/dL)	14
Mean Corpuscular Volume (79.0 - 94.8 fL)	94
Platelet Count (163 – 369 thousand/uL)	274

## Discussion

This case illustrates several reasons why G6PD deficiency may be diagnosed late in life. First, many individuals with G6PD deficiency remain asymptomatic unless exposed to significant oxidative stress. Patients who avoid classic triggers, either incidentally or due to lifestyle, dietary habits, or limited medication exposures, may never experience overt hemolytic crises. The commonly cited acute hemolysis symptoms of jaundice, fatigue, and back or abdominal pain are not seen in mild cases [1]. Second, mild or intermittent hemolysis may present as subtle anemia that is easily attributed to aging, chronic disease, nutritional deficiencies, or renal insufficiency, which are far more common in older adults.

Routine screening for G6PD deficiency is not standard practice in asymptomatic adults in many regions. Peripheral smear findings may be subtle or transient, and enzyme levels can be falsely normal during acute hemolysis due to reticulocytosis, further complicating diagnosis [5]. In older patients, clinicians may have a lower index of suspicion for inherited hemolytic disorders, contributing to diagnostic delay.

Worldwide, ingestion of fava beans is the most common trigger, followed by medication such as certain antibiotics, antimalarials, or sulfa-containing medications (Table 3) [2]. Recognition of G6PD deficiency is important because it allows the opportunity to educate patients to avoid triggers. There have been severe cases where ingestion of fava beans leads to blood transfusions and hospitalizations due to unawareness of G6PD deficiency, especially in children [6]. However, some cases can be severe without ever having a trigger identified [6]. Management of acute hemolysis due to G6PD deficiency is supportive as it is self-limited once the oxidative stressor is removed. G6PD activity declines as red blood cells age; thus, younger red blood cells with inherently higher G6PD activity levels survive, halting the hemolytic process. For this same reason, testing for G6PD deficiency during an episode of acute hemolysis, with brisk reticulocytosis, leads to false-negative results.

**Table 3:** List of common triggers for G6PD deficiency

Medication triggers
Chlorpropamide
Dabrafenib
Dapsone
Methylene blue
Nitrofurantoin, nifuratel, and nitrofurazone
Phenazopyridine
Primaquine and tafenoquine
Rasburicase and pegloticase
Sulfonylureas (such as glipizide, glyburide)
Food and chemical triggers
Fava beans
Henna
Naphthalene (moth balls, pesticides)

An additional and often underappreciated implication of diagnosing G6PD deficiency is its impact on the interpretation of glycated hemoglobin (HbA1c) values, which is commonly used to screen for and monitor diabetes mellitus. In patients with G6PD deficiency, intermittent or chronic low-grade hemolysis shortens red blood cell lifespan, leading to falsely low HbA1c measurements. This may delay the diagnosis of diabetes or result in underestimation of glycemic control, particularly in older adults who are already at increased risk for type 2 diabetes. When evaluated in the United Kingdom, it was found that male G6PD carriers were diagnosed with diabetes on average 4.1 years later than non-carriers [4]. In G6PD-deficient patients, fructosamine levels offer greater accuracy than HbA1c values.

## Conclusions

G6PD deficiency can remain undiagnosed until late adulthood in individuals who are not exposed to classic hemolytic triggers. This case underscores the importance of considering inherited red blood cell enzymopathies in the differential diagnosis of unexplained, intermittent anemia in older adults. Increased awareness may prevent iatrogenic hemolysis and improve patient safety through appropriate counseling and medication avoidance. Clinicians should consider alternative methods of glycemic assessment when fasting plasma glucose or oral glucose tolerance testing seem discordant with HbA1c values. 
